# Notochordal cell conditioned medium stimulates mesenchymal stem cell differentiation toward a young nucleus pulposus phenotype

**DOI:** 10.1186/scrt18

**Published:** 2010-06-16

**Authors:** Casey L Korecki, Juan M Taboas, Rocky S Tuan, James C Iatridis

**Affiliations:** 1 Cartilage Biology and Orthopaedics Branch, National Institute of Arthritis, Musculoskeletal and Skin Diseases, National Institutes of Health, Department of Health and Human Services, 50 South Drive, Bethesda, MD 20892, USA; 2 Center for Cellular and Molecular Engineering, Department of Orthopaedic Surgery, University of Pittsburgh School of Medicine, 450 Technology Drive, Pittsburgh, PA 15219, USA; 3 School of Engineering, University of Vermont, 33 Colchester Avenue, Burlington, Vermont 05405, USA

## Abstract

**Introduction:**

Mesenchymal stem cells (MSCs) offer promise for intervertebral disc (IVD) repair and regeneration because they are easily isolated and expanded, and can differentiate into several mesenchymal tissues. Notochordal (NC) cells contribute to IVD development, incorporate into the nucleus pulposus (NP), and stimulate mature disc cells. However, there have been no studies investigating the effects of NC cells on adult stem cell differentiation. The premise of this study is that IVD regeneration is more similar to IVD development than to IVD maintenance, and we hypothesize that soluble factors from NC cells differentiate MSCs to a phenotype characteristic of nucleus pulposus (NP) cells during development. The eventual clinical goal would be to isolate or chemically/recombinantly produce the active agent to induce the therapeutic effects, and to use it as either an injectable therapy for early intervention on disc disease, or in developing appropriately pre-differentiated MSC cells in a tissue engineered NP construct.

**Methods:**

Human MSCs from bone marrow were expanded and pelleted to form high-density cultures. MSC pellets were exposed to either control medium (CM), chondrogenic medium (CM with dexamethasone and transforming growth factor, (TGF)-β3) or notochordal cell conditioned medium (NCCM). NCCM was prepared from NC cells maintained in serum free medium for four days. After seven days culture, MSC pellets were analyzed for appearance, biochemical composition (glycosaminoglycans and DNA), and gene expression profile (sox-9, collagen types-II and III, laminin-β1 and TIMP1(tissue inhibitor of metalloproteinases-1)).

**Results:**

Significantly higher glycosaminoglycan accumulation was seen in NCCM treated pellets than in CM or TGFβ groups. With NCCM treatment, increased gene expression of collagen III, and a trend of increasing expression of laminin-β1 and decreased expression of sox-9 and collagen II relative to the TGFβ group was observed.

**Conclusions:**

Together, results suggest NCCM stimulates mesenchymal stem cell differentiation toward a potentially NP-like phenotype with some characteristics of the developing IVD.

## Introduction

The developing intervertebral disc (IVD) is formed as a result of an interaction between mesenchymal and notochordal (NC) cell types. For many organisms, NC cells disappear from the nucleus pulposus (NP) with aging and are replaced with chondrocyte-like cells, possibly suggesting an important functional role for NC cells during development. The age that NC cells disappear varies with animal species, although some species, such as pig, retain NC cells in maturity [1]. In humans, the loss of NC cells occurs at a very young age [2-5]. Disappearance of NC cells may contribute to matrix changes, typically characterized by a transition from a healthy gelatinous structure to a fibrous composition similar to that of the surrounding annulus fibrosus (AF) tissue, as well as decreases in cellularity during aging [6,7]. The NP tissue of NC cell retaining species is easily distinguished from that of non-NC cell retaining species by its higher proteoglycan and water content and a generally less fibrous nature [6].

NC cells have been morphologically described as being rounded, larger than NP cells, containing vacuoles, and with an extensive cytoskeletal network [1,8-10]. Recent studies have further defined the NC cell phenotype by successfully sorting them from the smaller NP cells using fluorescence-activated cell sorting (FACS) [7]. Although no specific marker(s) exists for the NC or NP cell types, their obvious size differences allow for successful separation by fluorescence-activated cell sorting based on side scatter and forward scatter parameters. The ability to separate the NC and NP cells also allowed for the first accurate comparisons of their respective gene expression profiles, and comparison to AF cells from the same animals [7].

Mesenchymal stem cells (MSCs) are adult tissue derived progenitor cells that exhibit clonogenicity, extended proliferative activity, and the ability to undergo multi-lineage differentiation [11]. The differentiation of MSCs into a chondrocyte phenotype through culture maintenance in a chondrogenic medium, specifically the supplementation of TGFβ, has been well documented [12]. A specific requirement for MSC chondrogenic differentiation *in vitro *is high cell density, for example in a three-dimensional pellet culture. While the phenotypic difference between chondrocytes and NP cells is a source of debate, it is clear that NP cells and chondrocytes share many common characteristics, such as expression of collagen type II and aggrecan, and it is likely that three-dimensional pellet culture is likewise a requirement for NP cell differentiation.

The current lack of specific marker(s) for IVD cells limits the ability to meaningfully explore the differentiation of MSCs to an IVD phenotype. In addition to studying MSC differentiation using more traditional chondrogenic markers of aggrecan, SRY-box 9 (SOX9), and collagen type II, this study sought to probe for expression of candidate markers referenced in the literature that together indicate a phenotype of cells unique to the IVD regions. In this study, we looked at three additional protein transcripts shown to be distinctly expressed in IVD tissues, collagen type III, laminin β1 and tissue inhibitor of metalloproteinases-1 (TIMP1). Collagen type III is the first collagen type expressed in the developing NP of the rat [13] and has been reported to be present in the NP and AF regions during both human and rat IVD development [14]. Another member of the laminin family, laminin γ1, is present in the developing NP and inner AF regions, with localization primarily peripheral in the early NP (E15/16) but present later in the NP and AF (E20) [13]. NP cells exhibit preferred adhesion to laminin γ1 coated culture plates relative to AF cells [15]. TIMP1 has been shown to be present in NC cell conditioned medium (NCCM) by mass spectroscopy [16] and is highly differentially expressed by NC, NP and AF cells [7].

The fundamental premise of this study is that IVD regeneration is more similar to IVD development than to IVD maintenance, with the corollary that NC cells are likely to be important contributors to the process of regeneration. The purpose of this study was thus to begin to investigate if NC cells may be used to recapitulate the developmental stages in the IVD by differentiating MSCs, a promising candidate cell type for tissue regeneration, to a phenotype with characteristics of the developing NP. As no phenotypic markers currently exist to distinguish NP cells from chondrocytes, we probed expression of some genes found to be present during IVD development in an attempt to distinguish MSC differentiation by TGFβ3 from that by NCCM. We hypothesized that NC cell conditioned medium will promote differentiation of MSCs toward a generally chondrogenic phenotype that is distinct from that induced using a TGFβ based chondrogenic medium, and with characteristics similar to NP cells. Should NCCM contain soluble factors that differentiate MSCs to a NP phenotype, the eventual clinical goal would be to isolate or chemically/recombinantly produce the active agent to induce the therapeutic effects, and to use it either as an injectable therapy for early intervention on disc disease, or in developing appropriately pre-differentiated MSC cells in a tissue engineered NP construct.

## Materials and methods

### Preparation of notochordal cell conditioned medium

The NP and AF regions from five porcine spines (approximately 10 intervertebral discs, from lower cervical, thoracic and lumbar regions as available, average age approximately two years) were separated from the surrounding tissue and cultured *in vitro *at 37°C and 5% CO_2 _in washing medium (modified from [17]) consisting of high glucose Dulbecco's Modified Eagle Medium (DMEM) with 10% fetal bovine serum (FBS), 100 μg/mL kanamycin, 200 U/ml penicillin and streptomycin, 0.50 μg/ml amphotericin-B (Sigma-Aldrich, St. Louis, MO, USA) and 0.5% v/v 5 M NaCl and 0.4 M KCl to adjust medium osmolarity to simulate the IVD environment. In general, medium was only supplemented with FBS to help cells recover from procedural steps and all medium used for conditioning of MSCs was serum free. After four days of culture to ensure no microorganism contamination was present, the tissue explants were separated by centrifugation and the supernatant was removed. The tissue was then pooled by tissue region, minced, and digested following a previously published protocol [17]. Pronase at 0.4% (w/v) for the AF or 0.2% for the NP (EMD Biosciences, Gibbstown, NJ, USA) was added to high glucose DMEM with 5% FBS for one hour with gentle agitation at 37°C, followed by four to six hours of digestion with collagenase type II at 0.2% for the NP, 0.4% for the AF (Worthington Biochemical, Freehold, NJ, USA) again in high glucose DMEM with 5% FBS. The resulting cell suspensions were passed through a 70 μm cell sieve and washed three times by centrifugation at 300 g with phosphate buffered saline (PBS).

FACS (fluorescence activated cell sorting) was used to separate the large NC cells from the small NP cells of the NP tissue fraction [7]. Due to the complexity of the FACS set-up, the tissue from the five pig spines was pooled for sorting. Both AF and NP cell suspensions were washed once with 1× non-enzymatic cell dissociation solution (Sigma-Aldrich, St. Louis, MO, USA). Cell suspensions were then centrifuged at 300 g, the supernatant was removed, and cells resuspended in FACS buffer (phosphate buffered saline, PBS, with 0.1% FBS) and placed on ice prior to FACS. The sorting protocol was based on a previously published protocol that analyzed forward scatter (FSC) and autofluorescence parameters [7]. The large NC cells, small NP cells and AF cells were retained and sorted into 100% FBS on ice.

Following sorting, all cell suspensions were pelleted by centrifugation, resuspended in medium (high glucose DMEM with 10% FBS, 100 U/ml penicillin and streptomycin, 0.25 μg/ml amphotericin-B, and 0.5% v/v 5 M NaCl and 0.4 M KCl) and plated for visual confirmation of sort efficacy (that is, only NC or NP cells in the correct category). The NC cells were then retained and incubated overnight in medium (high glucose DMEM with 10% FBS, 100 U/ml penicillin and streptomycin, 0.25 μg/ml amphotericin-B, and 0.5% v/v 5 M NaCl and 0.4 M KCl) at 5% O_2 _and CO_2_, 37°C to recover. The next day, cells were seeded into alginate gels at 2 × 10^6 ^cells/mL and cast into discs (height of 3.27 ± 0.15 mm and diameter of 5.82 ± 0.22 mm, using 100 μL of alginate-gel solution) using a slow set technique [18]. Alginate culture was used to maintain a three-dimensional culture environment for the NC cells.

NCCM was produced with five gels placed into each well of a six-well non-treated tissue culture plate and incubated in 5 mL of serum free medium (high glucose DMEM, 100 U/ml penicillin and streptomycin, 0.25 μg/ml amphotericin-B, and 0.5% v/v 5 M NaCl and 0.4 M KCl). Therefore, the starting cell density of the resulting conditioned medium NCCM was 1,000,000 NC cells per 5 mL medium. The cultures were maintained for four days. The nature of the NCCM was confirmed by examination of the cytomorphology and viability of NC cells following release from alginate. The retention of NC cell cytomorphology was confirmed by comparing released cells with images obtained before alginate embedding, and with descriptions of NC cells in the published literature [1,7]. Viability was assessed using the LIVE/DEAD assay (Molecular Probes, Eugene, OR, USA), where cells were incubated in 4 mM Calcein-AM and 2 mM Ethidium homodimer-1 in PBS to determine numbers of live and dead cells, respectively. The NCCM was then frozen and stored for future use by direct application to human MSCs derived from three different patients in three separate experiments as described below.

### Isolation and differentiation of human MSCs

Human MSCs were harvested with informed consent from the bone marrow of three patients undergoing total hip replacement surgery (University of Washington; mean age 64.3, range 56 to 73) as described previously [19,20]. Briefly, bone marrow was flushed from the bone with high glucose DMEM then centrifuged once to remove the fat containing layer. The remaining suspension was then layered on a Ficoll-Paque density gradient (Amersham, Uppsala, Sweden) to isolate the mononuclear cells and plated onto tissue culture polystyrene flasks. After 24 hours, cultures were washed with PBS to remove non-adherent cells and material. Adherent MSCs were culture-expanded to passage 3 or 4 in high glucose DMEM with 100 U/ml penicillin and streptomycin, 0.25 μg/ml amphotericin-B and 10% FBS.

MSCs cell pellets consisting of approximately 250,000 cells were created in 96-well conical bottom plates (Nunc, Rochester, NY, USA). Pellets were incubated overnight in serum-free control medium (CM: high glucose DMEM, 1% ITS + premix, 50 μg/mL L-ascorbic acid 2-phospate, 40 μg/mL L-proline, 100 mg/mL sodium pyruvate) after which they were assigned to one of three groups: NCCM (applied directly without dilution), CM, or TGFβ (CM with 10 ng/mL recombinant human TGF-β3 (R & D systems, Minneapolis, MN, USA) and 100 nM dexamethasone). For each group, the culture medium was changed at the beginning of the seven day experiment and on Day 3 of culture. Pellets were maintained at 37°C and 5% CO_2 _and at 5% O_2 _to replicate the low oxygen tension present in the IVD.

After seven days in culture, the cell pellets were harvested and analyzed for sulfated glycosaminoglycans (sGAG) using the Blyscan assay kit (Accurate Chemical, Westbury, NY, USA) and DNA content using the Picogreen assay (Molecular Probes, Eugene, OR, USA). Pellets were flash-frozen, sectioned, and stained with Alcian blue to visualize sGAG distribution and content. To evaluate the differentiation state of the cells in pellets, total RNA was extracted using Trizol (Invitrogen, Carlsbad, CA, USA) according to the manufacturer's instructions. The gene expression profile was analyzed using quantitative reverse transcription-polymerase chain reaction (qRT-PCR) using human gene-specific primers and SYBR Green fluorophore: Collagen type II (F - AAGAAGGCTCTGCTCATCCAGG, R - TAGTCTTGCCCCACTTACCGGT) and collagen type III (F - TCTTCCTGGTCTGGCTGGTA, R - TCCTCGATGTCCTTTGATGC), SOX9 (F - TGAAGAAGGAGAGCGAGGAG, R - GTCCAGTCGTAGCCCTTGAG), TIMP1 (F - TACACCCCCGCCATGG, R - GTCCACAAGCAATGAGTG) and laminin β1 (F - AAGGAAGACGGGAAGAAAGGG, R - TACAGTAGGGTTCGGGCTTGTG). mRNA transcripts were quantified and levels normalized to that of glyceraldehyde-1-phosphate dehydrogenase (GAPDH) (F - ATCAAGAAGGTGGTGAAGCAGG, R - TGAGTGTCGCTGAAGTCG). The results were also normalized to CM conditions, and Student's *t*-test was used to identify statistically significant differences between NCCM and TGFβ medium conditions (significance level of *P *< 0.05).

## Results

**Figure 1:**
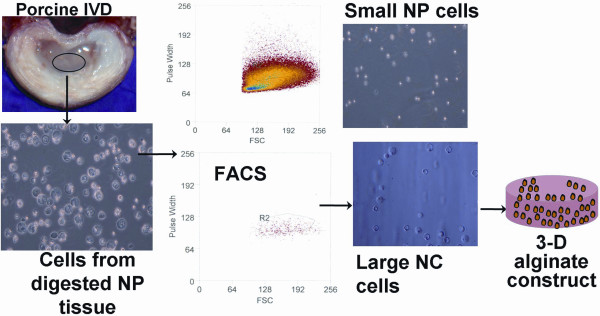
**Schematic of experimental design to produce NCCM**. NP tissue from the porcine IVD was harvested and digested to obtain a mixture of large NC and small NP cells. Large NC cells were isolated from the mixture by FACS (top FACS image is combined NC/NP cells with the reference gate to select NC cells, bottom FACS image is the isolated cells from the gate (NC cells). NC cells were embedded in alginate, and cultured for four days to produce conditioned medium (NCCM).

The sorting protocol was based on a previously published protocol that analyzed forward scatter (FSC) and autofluorescence parameters [7], with the exception that in our hands, autofluorescence was not found to be consistently distinguishable between small and large NP cells so this step was omitted. The AF cells were used as a reference *gate *population where pulse width and FSC were used to set the *gate*, followed by introduction of the NP/NC cell population and separation of the small NP cells (matching the reference *gate *population of the AF) and the large NC cells (larger pulse width and higher on average FSC parameter than the *gate *population) (Figure 1).

**Figure 2:**
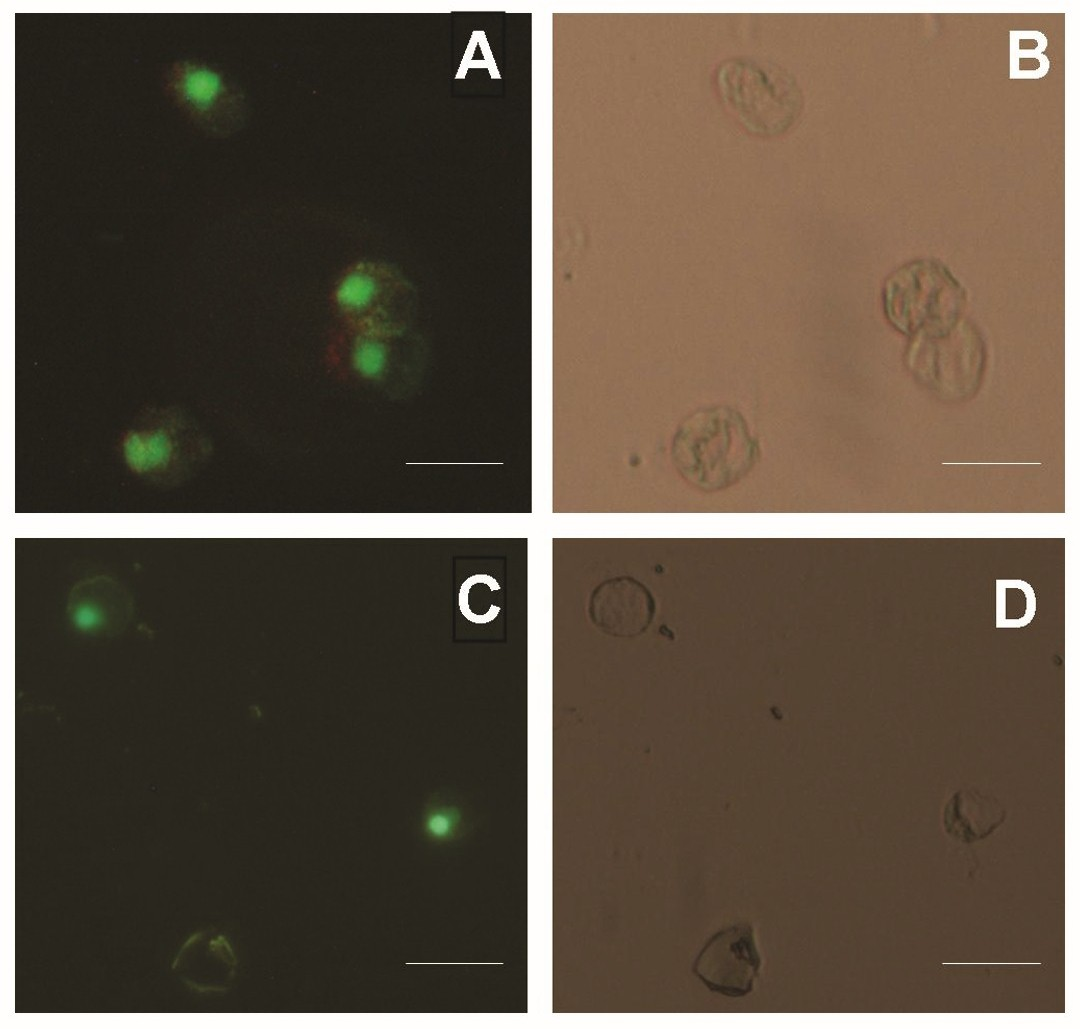
**Viability and morphology of NC cells embedded in alginate and cultured for four days**. Cell viability **(a, c) **was assessed using LIVE/DEAD staining and morphology **(b, d) **was observed by means of phase contrast microscopy. Cells retained viability and morphology after FACS (a, b) and after being embedded in alginate for four days (c, d). Cell viability dyes are calcein AM (green) and ethidium homodimer red (red). Scale bar = 200 microns.

After culture for four days in alginate, NC cells released from the alginate showed no decrease in viability or changes in morphological appearance (Figure 2a, b, c, d), supporting the use of alginate culture for future studies of NC cells. This is in contrast to NC cell morphology in monolayer, where the cells spread extensively and lose their morphological appearance of rounded cells with internalized vacuoles. These observations suggest that the NC cells retained their viability as well as phenotype in 3-D culture. Thus the NCCM represented a valid reagent to test the biological activity of secreted products of NC cells.

**Figure 3:**
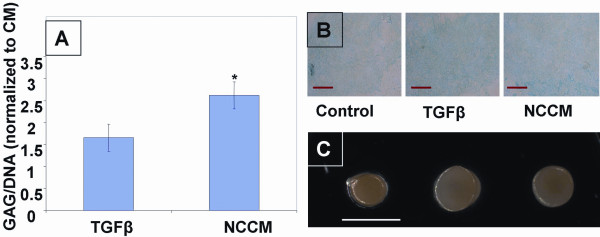
**Morphology and sGAG production in MSC pellet cultures treated with NCCM and TGFβ**. Cultures were examined after seven days of treatment. **A) **Significantly greater GAG production was noted in the NCCM than TGFβ group (* indicates significance, *P *= 0.04). Values are expressed as average ±SEM. sGAG/DNA normalized to CM group. **B) **Pellets stained with alcian blue and imaged at 20× show an increase in sGAG content with NCCM treatment. (L to R: CM, TGFβ medium, NCCM) Scale bar = 200 microns. **C) **NCCM and TGFβ treated pellets were larger than control pellets as analyzed by gross morphology (L to R: CM, TGFβ medium, NCCM) scale bar = 1.8 mm.

We next examined the effects of exposure to NCCM on the differentiation activities of human bone marrow MSCs in high density pellet culture as compared to TGFβ treatment. In terms of gross morphology, MSC pellets cultured in either TGFβ medium or NCCM were always visually larger compared to those cultured in CM, however the relative sizes of NCCM and TGFβ pellets varied between patients (Figure 3c). Biochemical analysis showed that GAG production (normalized to DNA content, which was not significantly different between groups) was enhanced in both NCCM and TGFβ cultures relative to CM cultures, with NCCM treatment significantly more effective than TGFβ treatment (10 ng/mL TGFβ3 and 100 nM dexamethasone) (Figure 3a). Culture in NCCM resulted in approximately 2.5 fold greater GAG accumulation than culture in CM, and also resulted in significantly greater accumulation of GAG than culture in TGFβ medium (*P *< 0.05). Histological images also showed more qualitative Alcian blue staining of sGAG in NCCM and TGFβ treated groups than in the CM group (Figure 3b).

**Figure 4:**
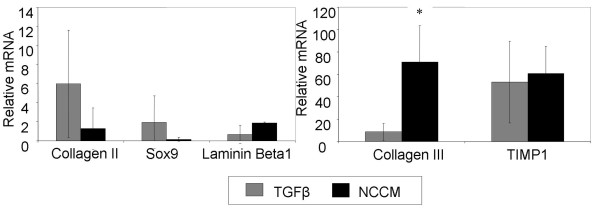
**qRT-PCR analysis of MSC pellet cultures treated with NCCM or TGFβ**. Expression of genes associated with chondrocyte or NP phenotypes were measured. Data were averaged over three patients, normalized to GAPDH and expressed relative to CM values (average +/- SEM). * indicates significantly greater collagen III expression in NCCM relative to TGFβ medium (*P *< 0.05).

NCCM and TGFβ had distinct effects on the expression levels of a number of genes relevant to chondrocyte and NP cell phenotype. We examined mRNA expression levels at a relatively early time point (Day 7 of culture) in order to assess and compare the early differentiation response of MSCs to both NCCM and TGFβ treated medium. Gene expression levels of collagen type II and SOX9, indicative of hyaline chondrocytes, were not upregulated as strongly in NCCM treated samples as TGFβ samples (Figure 4a). Gene expression for collagen type III was significantly up-regulated in NCCM versus TGFβ treated MSCs (*P *< 0.05) (Figure 4b). Laminin β1 gene expression tended to be higher in NCCM than TGFβ, however results were not statistically significant. No trends in TIMP1 expression were detected.

## Discussion

In this study we examined the possible trophic activity of NC cells by analyzing the effects of NCCM on the differentiation response of multipotent human MSCs in comparison to the response of MSCs to control medium or medium containing TGFβ and dexamethasone. Consistent with our hypothesis, NCCM promoted a phenotype in pellet cultures of MSCs that was distinct from that observed in cultures maintained in TGFβ and dexamethasone containing medium. Phenotype characteristics of NCCM-treated cells that are reminiscent of IVD development include a significant upregulation of collagen type III mRNA expression, a trend of increased laminin β1 mRNA expression, and significantly higher level of sGAG production than for TGFβ treated cells. In addition, NCCM conditioning of MSCs tended to up-regulate collagen type II mRNA less strongly than with TGFβ. Taken together, these results highlight the differences between the NCCM-induced phenotype and the chondrogenic phenotype induced by TGFβ, and suggest that NCCM has potential to differentiate MSCs towards a young NP phenotype.

A rigorous distinction of IVD cells from chondrocytes does not currently exist, despite evidence of phenotypic differences [21], due to a lack of unique cell surface markers or other definitive cellular characteristics. Therefore, with current scientific knowledge, NC cells and NP cells must be characterized morphologically and based on multiple protein and gene measurements. In an attempt to assess the possible IVD characteristics of NCCM-treated MSCs, expression levels of collagen type III (shown to be expressed early in NP tissue development [13]), laminin β1 (also found during early NP tissue development [13] and in the NP and inner AF of immature porcine spines [15]), and TIMP1 (shown to be differentially expressed in NC cells versus NP and AF cells [7]) were examined in this study in addition to the typical chondrocyte markers of SOX9 and collagen type II. Collagen type III clearly has a role in IVD development as it has been reported to be present in outer AF, inner AF, and NP regions during human [19] and rat IVD development [13]. It is the first collagen type expressed in the developing NP of the latter [13]. Immunoreactive collagen type III is strongly detected in the outer and inner AF regions in the neonate [13] finally localizing pericellularly in the outer AF [22]. However, increased collagen type III has also been reported pericellularly in degenerating discs [23] so that it may also be present during attempted repair or other degenerative processes. While increased collagen type III expression was observed by qRT-PCR in this study, no collagen type III was detected using immunohistochemical staining (data not shown), which could be the low level of protein accumulation during the time course used here. Laminin chains have been reported to be present in the developing and functional IVD. Laminin γ1 is present in the developing NP and inner AF regions, with localization primarily in the early NC (E15/16), but present later in the NP and AF (E20) [13]. NP cells attach to laminin γ1 coated plates in higher numbers than AF cells, indicating a cell phenotype-dependent role of this molecule in cell-matrix attachment [15]. Although no statistically significant increase in the expression of another member of the laminin family (laminin β1) was observed in NCCM-treated MSC pellet cultures, a trend of increasing expression was observed. Future work should examine the presence of other laminin chain isoforms, particularly as the role and importance of these extracellular matrix molecules is clarified. TIMP1 has also been shown to be highly differentially expressed by NC, NP and AF cells [7] and has been shown to be present in NCCM by mass spectroscopy analysis [16]. However, no difference between the expression levels of TIMP1 was observed between MSCs exposed to NCCM or TGFβ medium in this study.

Our results demonstrate that NCCM and TGFβ have compellingly different capacities to differentiate MSCs along chondrogenic pathways, and with notable distinctions. This study provides a first step for exploring how NC cells may contribute to future strategies for MSC based IVD regeneration. Yet some limitations should be considered that require additional investigation, particularly with respect to the specific mechanisms of action. For example, it is possible that NC cell secreted sGAG may have been present in the NCCM conditioned medium and been delivered to the MSC pellets. This may have contributed to the sGAG measured in the NCCM treated group; however, the pellets for the NCCM group always appeared large, suggesting that this would at most account for only a partial effect. The absence of a control cell conditioned medium group (such as a fibroblast conditioned medium, or conditioned medium from other cells of the IVD such as AF or NP cells), while not affecting the comparison between NCCM and chondrogenic medium treated cells, limits identification of unique contributions of NP cells to MSC differentiation. In addition to the production of soluble factors, NC cells could also have depleted some nutrients or essential factors in the NCCM. Taken together, our results highlight the potential for NCCM to stimulate MSC differentiation, and future investigations are required to establish mechanisms of these effects including longer duration studies and the addition of hypertrophic markers.

Previous studies have illustrated the stimulatory effects of NC cell conditioned medium on native IVD cells and chondrocytes [8,16,24,25]. Mass spectroscopic analysis has shown the presence of connective tissue growth factor (CTGF) in NCCM [24], suggesting a mechanism for the NC cell mediated change in IVD and chondrocyte cell metabolism. CTGF has been shown to be an important factor in cell proliferation and matrix remodeling during chondrogenesis [26], motivating future investigation along with other possible factors responsible for IVD cell specific differentiation.

## Conclusions

This study demonstrates that NC cells secrete soluble factor(s) that stimulate MSC differentiation down a lineage different from standard mesenchymal chondrogenesis. Specifically, NCCM induced significantly more collagen type III expression and sGAG production in MSCs, which when taken in the context of IVD development, may be an indication that NCCM induces a more NP-like phenotype than chondrogenic medium containing TGFβ3 and dexamethasone. Thus, we propose that NC cells may serve to maintain the young NP phenotype of IVDs in species that retain NC cells, and that this characteristic makes NC cells a promising target cell type to formulate approaches for IVD repair that may have similarities to development, and the potential for NCCM to influence MSC differentiation offers promise. While further research is needed to define an *NP phenotype *versus a *chondrocyte phenotype *before definitive conclusions can be drawn as to the differentiation profile of the MSCs, collagen III and laminin isoforms may provide useful markers in this distinction. Determining the mechanisms responsible for the NCCM effect will be critical in developing MSC based therapies for IVD repair.

## Abbreviations

AF: annulus fibrosus; CM: control medium; FACS: fluorescence-activated cell sorting; FBS: fetal bovine serum; FSC: forward scatter; IVD: intervertebral disc; MSC: mesenchymal stem cell; NC: notochordal; NCCM: notochordal cell conditioned medium; NP: nucleus pulposus; PBS: phosphate buffered saline; SOX9: SRY-box 9; TIMP1: tissue inhibitor of metalloproteinases-1

## Competing interests

The authors declare that they have no competing interests.

## Authors' contributions

CK performed cell culture experiments, participated in the design of the study and drafted the manuscript. JT helped in the design of the study and helped draft the manuscript. RT participated in the design and coordination of the study, and reviewed the manuscript. JI conceived of the study, participated in the design and coordination, and helped draft the manuscript.
